# MM/GBSA prediction of relative binding affinities of carbonic anhydrase inhibitors: effect of atomic charges and comparison with Autodock4_Zn_

**DOI:** 10.1007/s10822-023-00499-0

**Published:** 2023-03-17

**Authors:** Mackenzie Taylor, Junming Ho

**Affiliations:** School of Chemistry, The University of New South Wales, Sydney, NSW 2052 Australia

**Keywords:** MM/GBSA, Carbonic anhydrase inhibitors, Binding free energy, Molecular docking, Molecular dynamics simulations, Density functional theory, Atomic charge schemes

## Abstract

Carbonic anhydrase is an attractive drug target for the treatment of many diseases. This paper examines the ability of end-state MM/GBSA methods to rank inhibitors of carbonic anhydrase in terms of their binding affinities. The MM/GBSA binding energies were evaluated using different atomic charge schemes (Mulliken, ESP and NPA) at different levels of theories, including Hartree–Fock, B3LYP-D3(BJ), and M06-2X with the 6–31G(d,p) basis set. For a large test set of 32 diverse inhibitors, the use of B3LYP-D3(BJ) ESP atomic charges yielded the strongest correlation with experiment (R^2^ = 0.77). The use of the recently enhanced Autodock Vina and zinc optimised AD4_Zn_ force field also predicted ligand binding affinities with moderately strong correlation (R^2^ = 0.64) at significantly lower computational cost. However, the docked poses deviate significantly from crystal structures. Overall, this study demonstrates the applicability of docking to estimate ligand binding affinities for a diverse range of CA inhibitors, and indicates that more theoretically robust MM/GBSA simulations show promise for improving the accuracy of predicted binding affinities, as long as a validated set of parameters is used.

**Graphical abstract:**

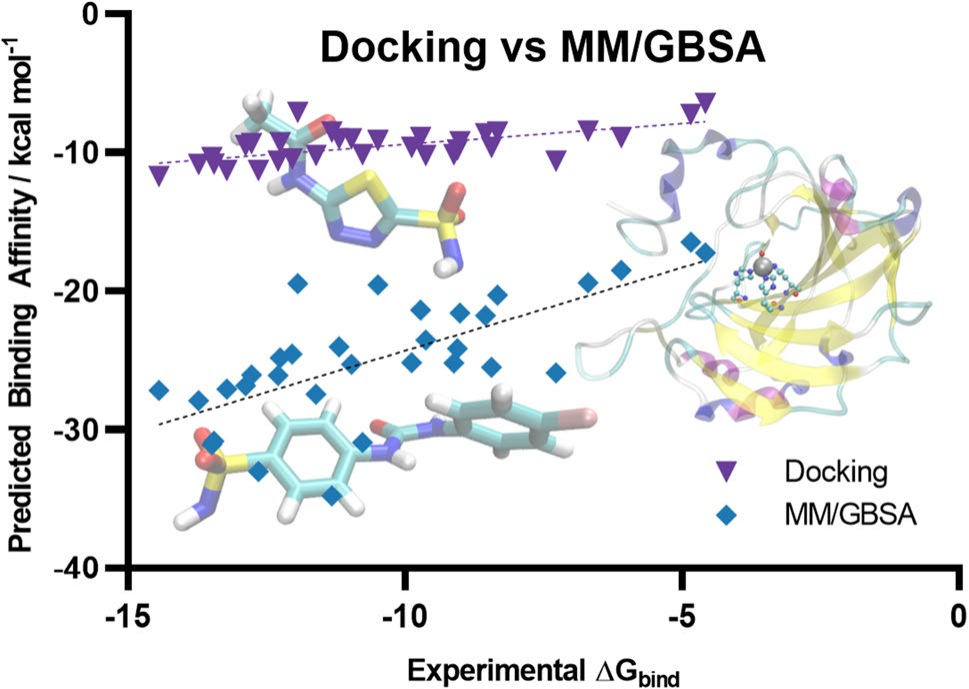

**Supplementary Information:**

The online version contains supplementary material available at 10.1007/s10822-023-00499-0.

## Introduction

The carbonic anhydrase (CA) superfamily of metalloenzymes is a class of enzymes which catalyse the reversible hydration of carbon dioxide. Fifteen isoforms with high structural similarity are found in humans, playing a role in CO_2_ transport, pH regulation, lipogenesis, gluconeogenesis, and ureagenesis [18, 68]. Different human CA (hCA) isoforms have been linked to a wide range of diseases including glaucoma and osteoporosis (hCA II), obesity (hCA VA and VB) and various cancers (hCA IX and XII) [8, 4, 10, 15, 41, 61, 69]. Consequently, designing CA inhibitors (CAIs) is of significant interest. To date, more than twenty-five drug compounds have been used clinically, with multiple others in clinical trials [45].

**Fig. 1:**
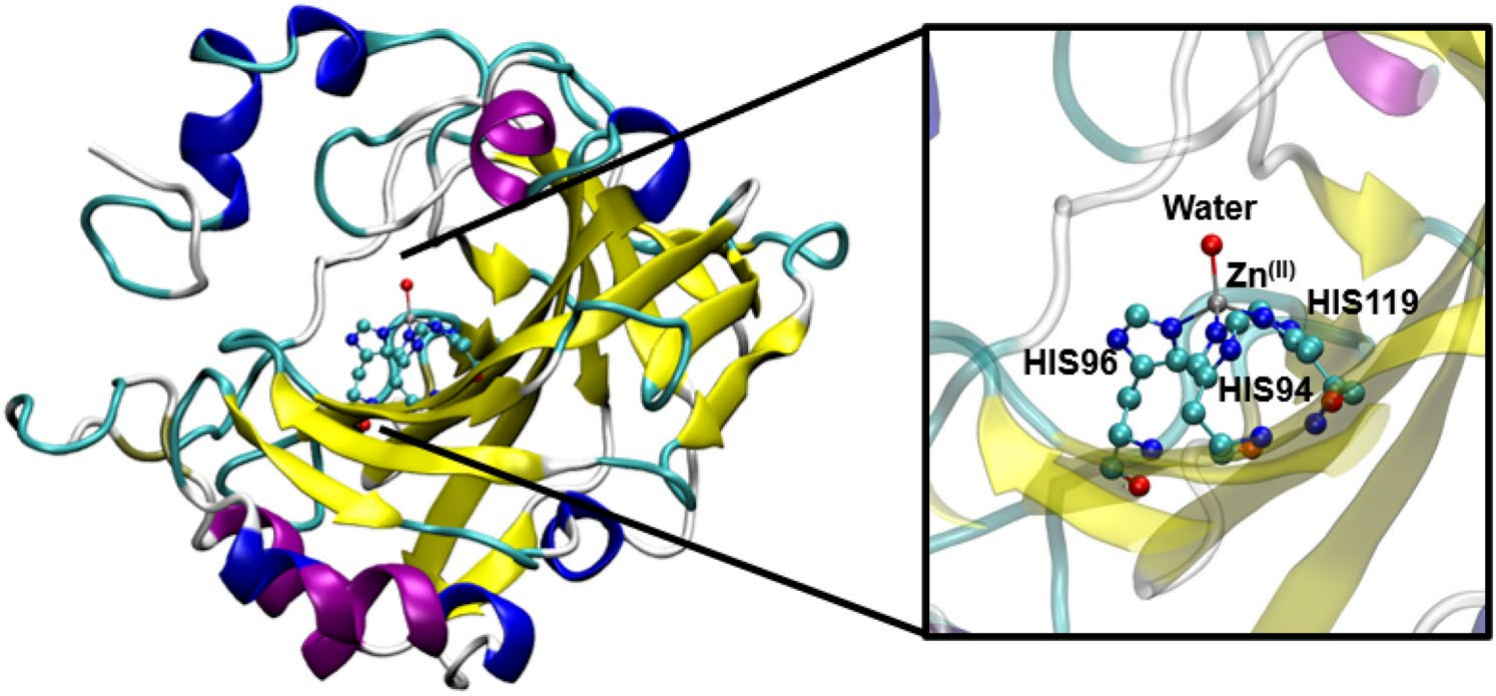
Structure of hCA II (PDB ID 2CBA). Protein backbone coloured by secondary structure. Purple represents α-helix, Blue represents a 3_10_ helix, yellow represent an extended β-sheet, white represents a coil, and brown represents a β-bridge

The general structure of hCAs is shown in Fig. 1. The active sites of all hCAs contain a Zn(II) ion that is coordinated to three histidine residues and a hydroxyl molecule in a tetrahedral arrangement [36]. The active site cavity has a narrow conical shape, giving a well-defined binding pocket. It is widely accepted that the hydration of CO_2_ is triggered by proton transfer from the Zn(II)-bound water to H64 to form a hydroxide anion followed by a nucleophilic attack of CO_2_ to form bicarbonate ion [3, 31].

The most common form of hCA inhibition involves a ligand replacing the coordinating water and binding directly to the zinc(II) ion. In particular, sulfonamide groups are widely used as the zinc-binding head group of CA inhibitors [70]. Clinical antiglaucoma agents such as acetazolamide, and brinzolamide contain a sulfonamide head group connected to a heteroaromatic ring structure, and bind at low nanomolar concentrations to several isoforms [57, 76]. Similarly, arylsulfonamides such as the ureido-substituted arylsulfonamide SLC-0111 show excellent potency when binding to hCA IX, with low nanomolar inhibition constants. This compound is currently in stage II clinical trials for the treatment of metastatic hypoxic cancers [46, 54].

A major challenge in targeting hCAs is posed by the high degree of structural similarity between the different isoforms [58]. As a result, selective inhibition of individual isoforms is very challenging. Given the broad distribution, and the wide range of applications of different hCA isoforms throughout the body, the use of non-selective inhibitors can lead to off-target binding. One example of this is the drug acetazolamide, which has been shown to have applications for glaucoma, altitude sickness, and as a seizure medication, but has fallen out of clinical use due to the number of side effects [8, 35, 61]. Consequently, the design of novel hCA inhibitors must consider both the potency and the selectivity of the ligand. Generally, this selectivity is gained through appending chemical groups to the ligand which reach into the more chemically diverse outer region of the binding site [7, 49, 71]. For this reason, it is important to develop tools which can accurately model inhibitor binding affinity both within and between isozymes.

Molecular docking, molecular dynamics (MD) simulations, and more recently the use of machine learning are commonly used to predict protein–ligand binding energies. For example, molecular docking is often used to predict binding poses and for pre-screening large libraries of compounds [13, 19, 20, 63, 72, 81]. In the context of CA inhibitor design, molecular docking has been widely used to screen ligands against a variety of isoforms [17, 27, 30, 38]. Recently, a docking protocol was used to screen a library of compounds against CA VII and identified four ligands with low nanomolar potency [23]. However, the predictive power of docking methods for screening hCA ligands was limited until a scoring function (Autodock4_Zn_) was optimised to better describe the interactions between the ligand and the Zn(II) centre [64]. One recent assessment study of seven docking programs indicated this approach had the highest scoring and ranking powers for a set of 97 zinc metalloenzymes ligand complexes [16].

More theoretically robust but computationally intensive MD studies of the energetics of CA binding are less common. Rossi and co-workers used the AMBER forcefield and free energy perturbation to predict the relative binding affinities of three ligands to hCA II within 1 kcal mol^−1^ of experimental values [62]. In another study, a full reaction profile of acetazolamide binding to hCA II was acquired via steered molecular dynamics simulations, and the resulting binding affinity was in good agreement with experiment [77]. As an alternative, the MM/GBSA (or PBSA) approaches are relatively efficient MD-based methods for estimating binding affinities [78]. These ‘end-state’ methods calculate the binding energy based on the strength of the intermolecular interactions between the protein and ligand using configurations sampled from the MD trajectory. Solvent effects are accounted for in an implicit manner, with either a Poisson-Boltzmann (MM/PBSA) or generalized born (MM/GBSA) model. The non-electrostatic component of the solvent is recovered from the solvent accessible surface area.

MM/GBSA has previously been applied to the problem of ligand binding affinities of CAIs with some success. Guimarães used the OPLS-2005/TIP4P forcefields to compare the ability of MM/GBSA and two FEP-based approaches, Desmond and MCPRO +, to predict trends in the binding of a set of 13 simple substituted arylsulfonamides to hCA II. This study used default OPLS-AA charges, which are optimised to reproduce empirical properties such as heat of vaporisations and solvent densities. The MM/GBSA approach was shown to have a moderately strong correlation score of R^2^ = 0.60, and its performance was comparable with FEP approaches [28]. Further, work in the Meuwly group demonstrated that MM/GBSA binding energies yielded moderate correlation with experimentally derived binding free energies (R^2^ = 0.55) for a set of 17 diverse sulfonamide ligands [65].

Finally, our recent work examined whether the inclusion of a p*K*_*a*_ correction in MM/GBSA simulations improves their correlation with experiment. This is because experimental binding constants take into account the deprotonation of the sulfonamide ligand whereas this energetic cost is neglected in classical MD simulations since the ligand is modelled in its deprotonated form. That study concluded that the inclusion of this p*K*_*a*_ correction did not result in a noticeable change in the correlation with experimental data presumably because the uncertainty in MM/GBSA predictions are higher than these corrections [32].

In light of the promising performance of MM/GBSA as well as the recent improvements made to docking programs such as the extension of a zinc-optimised forcefield in Autodock Vina, this paper aims to address the following questions with the view to identifying effective protocols for prediction of *relative* CA-inhibitor binding energies:

1. Does the use of computationally expensive MD methods improve the prediction of the relative binding affinities compared to molecular docking?
2. Is it possible to further optimise the performance of MM/GBSA? Specifically, the performance of these methods is highly sensitive to the choice of atomic charges used to describe the electrostatic interactions between the enzyme and ligand [79]. For example, Guimarães employed empirically optimised OPLS charges while Meuwly used DFT derived Mulliken charges. In this paper, we sought to identify an optimal approach (level of theory and charge calculation scheme) that will maximise the correlation between MM/GBSA with experimental binding constants when considered across a diverse dataset.

**Fig. 2:**
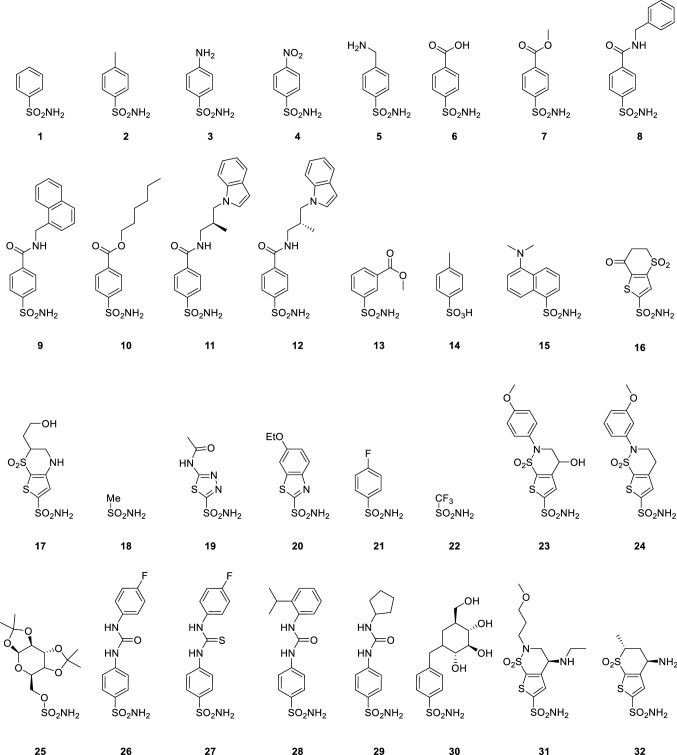
Dataset of CA II inhibitors with experimental binding affinities

Towards this end, we have assembled a dataset of 32 chemically diverse zinc-binding hCA inhibitors with experimentally measured binding free energies in hCA II (Fig. 2) [11, 37, 42, 53, 54, 65]. The first 15 ligands consist of arylsulfonamides studied in the Meuwly group [65]. In that study, two para-substituted arylsulfonamides were shown to be unstable during the MD simulations, and as such were not included here. Ligands **16**–**25**, and **30**–**32** are a set of more chemically diverse binding groups, containing multiple unique heterocycles. Finally, **26** is ureidobenzenesulfonamide (SLC-0111) [46] that is currently in clinical trials as an anti-cancer agent, and **27**-**29** are related derivatives [13]. This test set is used to evaluate the performance of the zinc optimised AD4_Zn_ forcefield used in conjunction with the Autodock Vina program as well as MM/GBSA calculations employing different ligand atomic charges obtained from Mulliken, electrostatic potential (ESP) and natural population analysis (NPA) schemes calculated at various levels of theory, viz. Hartree Fock, B3LYP-D3(BJ), and M06-2X. These atomic charge calculation schemes are chosen because they are used in popular force fields and/or used in previous MD simulations of CA-inhibitor complexes [43, 65].

## Computational details

### Molecular docking

Docking was performed with the AutoDock Vina program with the zinc metalloenzyme optimised AutoDock4_Zn_ [64]. The three dimensional protein structure (PDB ID: 1LUG, resolution 0.95 Å) [5] was obtained from the RCSB PDB database [6]. Ligand structures were obtained from crystal structures, or built in IQmol [25], and polar hydrogens were added in the AutoDock Tools suite [51]. Ligand structures were prepared with the Meeko package for docking [47] and docked poses were visualised with the Autodock Tools suite. The docking was conducted in triplicate with an exhaustiveness parameter of 32.

### Molecular dynamics simulations

The starting structure of hCA II was obtained from the crystal structure (PDB ID: 1LUG). Protein protonation state was determined with the H +  + server [26] at a pH of 6.5 and a salinity of 0.15 M, and missing hydrogens were added by this server. Ligands were protonated with the AmberTools “Reduce”program [12]. One sulfonamide hydrogen was manually removed to form the ligand in its anionic state. The ligand was then cleaned with the pdb4amber function in AmberTools. TIP3P water molecules [33] were added to create a border of 8 Å with the solvate function in VMD, resulting in a box size of 63 × 60 × 71 Å. Counterions were added to maintain electroneutrality via the “autoionize” VMD plugin. Initial ligand configurations were obtained from crystal structures where available, or through docking with the Autodock Vina program using the AutoDock4_Zn_ forcefield (ligands** 9**, **10**, **13**, **14**, **16–18**).

Simulations were conducted in NAMD 2.13 [55, 56] with periodic boundary conditions at a constant temperature of 300 K. The Langevin algorithm was used to keep a constant pressure of 1 bar with the Noose-Hoover Langevin Piston method using a timestep of 2 fs. The Particle Mesh Ewald algorithm [80] was used to calculate long range electrostatics, with a distance cut-off of 12 Å. The bonds of the TIP3P water molecules were kept rigid using the RATTLE algorithm. Harmonic restraints were placed on the backbone of the protein with a force constant of 2 kcal mol^−1^ Å^−1^. The system was minimized for 10 000 steps and heated to 300 K over 400 ps. The harmonic restraints were then gradually reduced to 0.1 kcal mol^−1^ Å^−1^ over 2.5 ns. The system was subsequently equilibrated for 4 ns, and production runs were carried out for 4 ns. For each ligand, 5 independent trajectories were generated. The CHARMM protein [9] and CGenFF [73] forcefields were used to model the complex. Ligand atom types and bonded parameters were assigned from the CGenFF server [74, 75]. For the sulfonamide ligands, the N–S–O angle was set to 111.00° with a force constant of 80 kcal mol^−1^ rad^−2^ to better reproduce the sulfonamide geometry.

### MM/GBSA

In MM/GBSA, the free energy of a system is estimated from Eq. **1**

1 $$\Delta G = E_{bond} + E_{el} + E_{vdw} + G_{polar} + G_{npolar} - TS$$

where *E*_*bond*_*, E*_*el*_*,* and *E*_*vdW*_ correspond to the standard MM bonded, electrostatic, and vdW energy terms. *G*_*polar*_ and *G*_*npolar*_ are the polar and non-polar contributions to the solvation free energy respectively. The final term is the temperature *T* multiplied by the entropy *S*, estimated from a normal-mode analysis or quasi-harmonic approximation approach. For the change in free energy as the free ligand (L) binds to the protein (P) is typically computed through Eq. 2

2 $$\Delta G_{bind} = \left\langle {G_{P - L} - G_{P} - G_{L} } \right\rangle_{PL}$$

Here, the binding affinity is evaluated as the difference in the energy between the protein—ligand complex **PL**, and the isolated protein **P** and ligand **L**, averaged over all configurations sampled from the MD simulation of the protein–ligand (PL) complex. The inclusion of entropic effects is typically the most computationally intensive part of the MM/GBSA analysis. Consequently, this effect is often neglected in MM/GBSA calculations. Meuwly’s work demonstrated the inclusion of entropy did not improve the prediction of trends in binding affinities for hCA inhibitors [65] and hence it is not included in this work. Therefore, the binding energy is calculated from Eq. 3.

3 $$\Delta E_{MM} + \Delta G_{S} = \left\langle {(E_{gas}^{PL} + G_{polar}^{PL} + G_{npolar}^{PL} ) - (E_{gas}^{P} + G_{polar}^{P} + G_{npolar}^{P} ) - (E_{gas}^{L} + G_{polar}^{L} + G_{npolar}^{L} )} \right\rangle_{PL}$$

To save on computational cost and to reduce the noise in the calculations, it is common that each term is evaluated on frames from the trajectory of the bound complex (indicated by <  > _PL_) [24, 34]. Hence, the reorganisation energy needed to change the conformational state of the unbound protein and ligand are also not considered.

The polar and non-polar contributions to the solvation free energy is calculated using a Generalized Born solvent model and consideration of the solvent accessible surface area [50] MM/GBSA energies were evaluated with the MMPBSA.py script in the AmberTools21 package [12, 29, 44]. Frames were sampled at 20 ps intervals from 4 ns production runs, as the binding affinity was found to have converged by this point. A salt concentration of 0.15 M was specified for the MM/GBSA calculations.

### QM calculation of atomic charges

QM calculations were performed using the Gaussian16 package [22]. Ligand geometries were optimised from their bound pose with either HF, M06-2X, or B3LYP-D3(BJ). All calculations were performed with the 6-31G(d,p) basis set. ESP charges from the Merz-Kollman scheme [66], Mulliken [52], and NPA charges [60] were evaluated from the optimised geometry at the corresponding level of theory in the gas phase. As recommended in previous MD studies [43, 65], the atomic charge for the Zn atom was set to + 1, in close agreement to the charge from a B3LYP-D3(BJ)/6-31G(d,p) calculation of a cluster containing a zinc, arylsulfonamide **1**, and three methylimidazole rings. The zinc atomic charge was found to be 0.914 (Mulliken), 0.643 (ESP), 1.258 (NPA).

## Results and discussion

### Docking of ligands with Autodock Vina

**Fig. 3:**
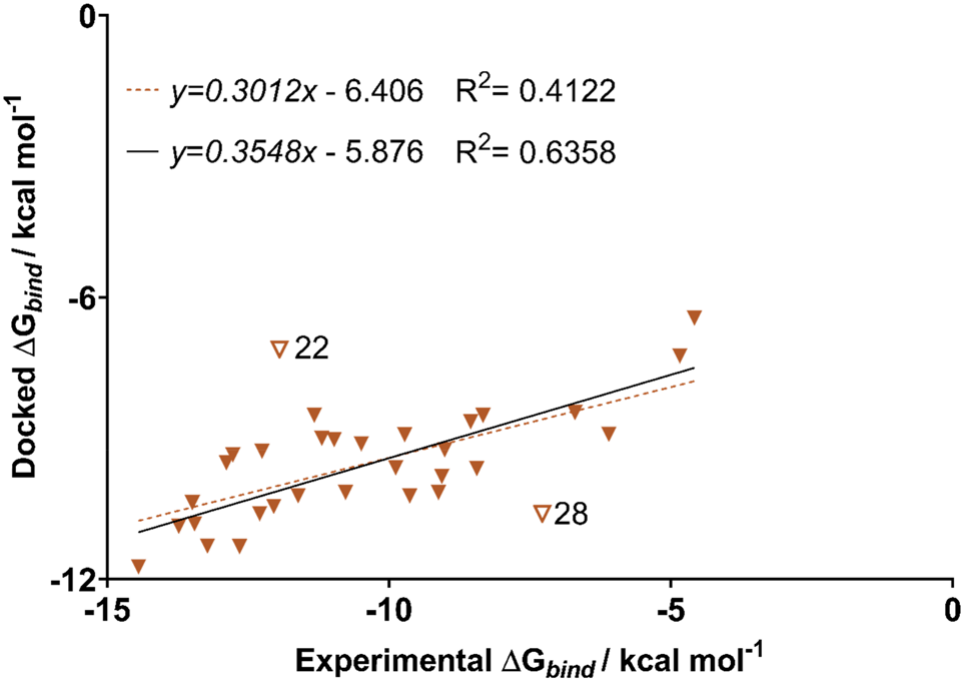
Predicted binding affinity of the full dataset of ligands by docking with the AutoDock4Zn force field in AutoDock Vina. Trendline shown with all data points (dashed) and with outliers removed (solid). Outliers denoted with a hollow symbol

To address the first question posed in the introduction, we have employed the AutoDock4_Zn_ force field and Autodock Vina program to predict the binding affinities of the test set of 32 ligands docked in their deprotonated form. In a recent assessment study, this docking program was found to perform very well for zinc metalloenzymes [16]. In this study, we see similarly good performance for the test set of 32 ligands where the correlation between the predicted and experimental binding affinity is shown in Fig. 3.

When the entire dataset is considered, Autodock Vina yielded a relatively weak correlation with experimental binding affinities (R^2^ = 0.41). However, if two notable outliers, ligands **22** and **29** were removed, the correlation is significantly improved (R^2^ = 0.64). Ligand **22** has an experimental p*K*_*a*_ of 6.3 [37] which is about 2–4 p*K*_*a*_ units lower compared to most of the other ligands. Including the effect of deprotonation is expected to increase the binding energy of **22** by approximately 2 to 5 kcal mol^−1^ (more negative) relative to the other ligands which would bring it closer to the trend line. Indeed, Figure **S1** in the Supporting Information shows that for ligands where experimental p*K*_a_ values are available, the inclusion of the p*K*_a_ correction in the docking scores improves the correlation with experimental values. Interestingly, **28** is structurally very similar to **26**, **27** and **29**, however it is the only one of the four analogues that is an outlier.

**Fig. 4:**
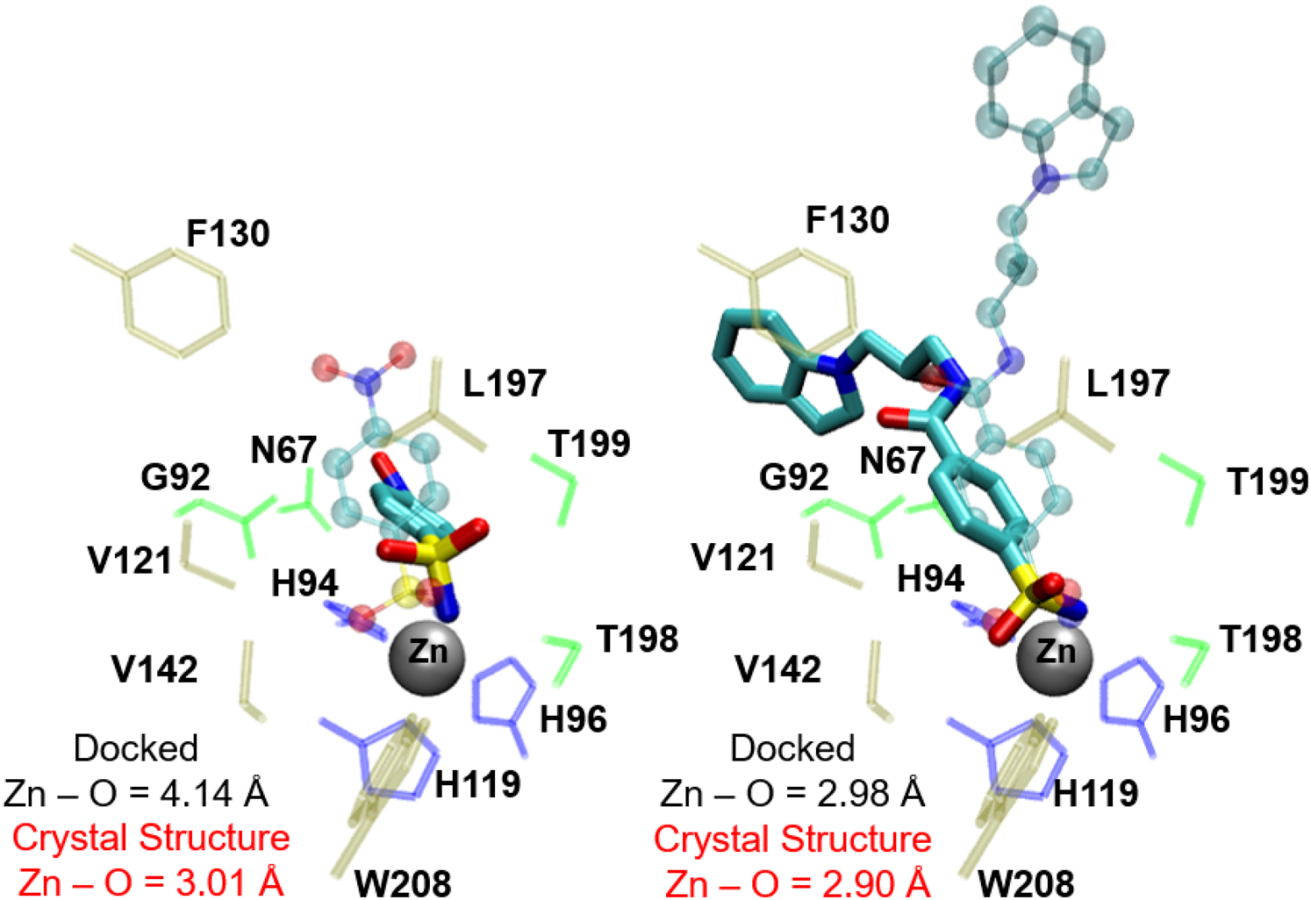
Comparison of crystal and docked binding poses of arylsulfonamide ligands **4** (left) and **11** (right). The crystal structure pose is shown in translucent stick and ball, and the docked pose is shown as full colour liquorice. Protein residues shown coloured by their residue type (non-polar residues in brown, polar residues in green, acidic residues in blue). Zn-sulfonamide oxygen (Zn-O) distances in the docked poses and crystal structures are also displayed

Despite the reasonably good correlation between the docking scores and experimental binding energies, there was a consistent difference between the predicted bound pose of the ligands compared to crystal structures. For the 24 ligands with crystal structures, the average RMSD between the crystal structure pose and lowest energy docked pose is > 6 Å. No ligands had a lowest energy binding mode with an RMSD of less than 2.5 Å from the crystal structure—in one case, **25** is predicted to bind outside of the active site. Two example ligands, arylsulfonamides **4** and **11** are shown in Fig. 4. In the crystal structures of their complexes with hCA II, the sulfonamide oxygen is also in close proximity to the Zn(II) ion (ca. 3 Å) which resembles a bidentate binding mode. When **4** is docked into the protein, the predicted binding pose is unable to correctly model the bidentate zinc—ligand interaction where the Zn–O distance is about 1 Å larger than in the crystal structure. As a result, the orientation of the phenyl ring in the docked pose and crystal structure are significantly different. On the other hand, **11** is predicted to have the correct bidentate zinc coordination, however the position of the long tail is incorrect. When the docked poses with the smallest deviation from the crystal structure is considered regardless of the corresponding binding energy, the mean RMSD reduces to 4.30 Å. Ligand **3** has the lowest RMSD of 1.9 Å, for a binding mode 0.23 kcal mol^−1^ higher in energy than the lowest energy pose. However, for 4 ligands—**11**, **12**, **23** and **24**, the AutoDock Vina program is unable to predict a docked pose within 6 Å of the crystal structure. When the energy of the binding pose with the smallest deviation from the crystal structure is used, no correlation with experiment is observed (R^2^ = 0.09).

### Validation of bonded parameters to maintain the zinc coordination geometry

In MD simulations of ligand binding to hCAs, it is important to preserve the tetrahedral coordination environment around Zn(II). Previously, both “bonded” [1, 40, 43] and “non-bonded” [67] approaches have been used to retain the tetrahedral zinc coordination. In the former, force field parameters are developed to model the Zn-ligand and Zn-Histidine bonds while in the latter, no bonded parameters are defined for Zn.

**Fig. 5:**
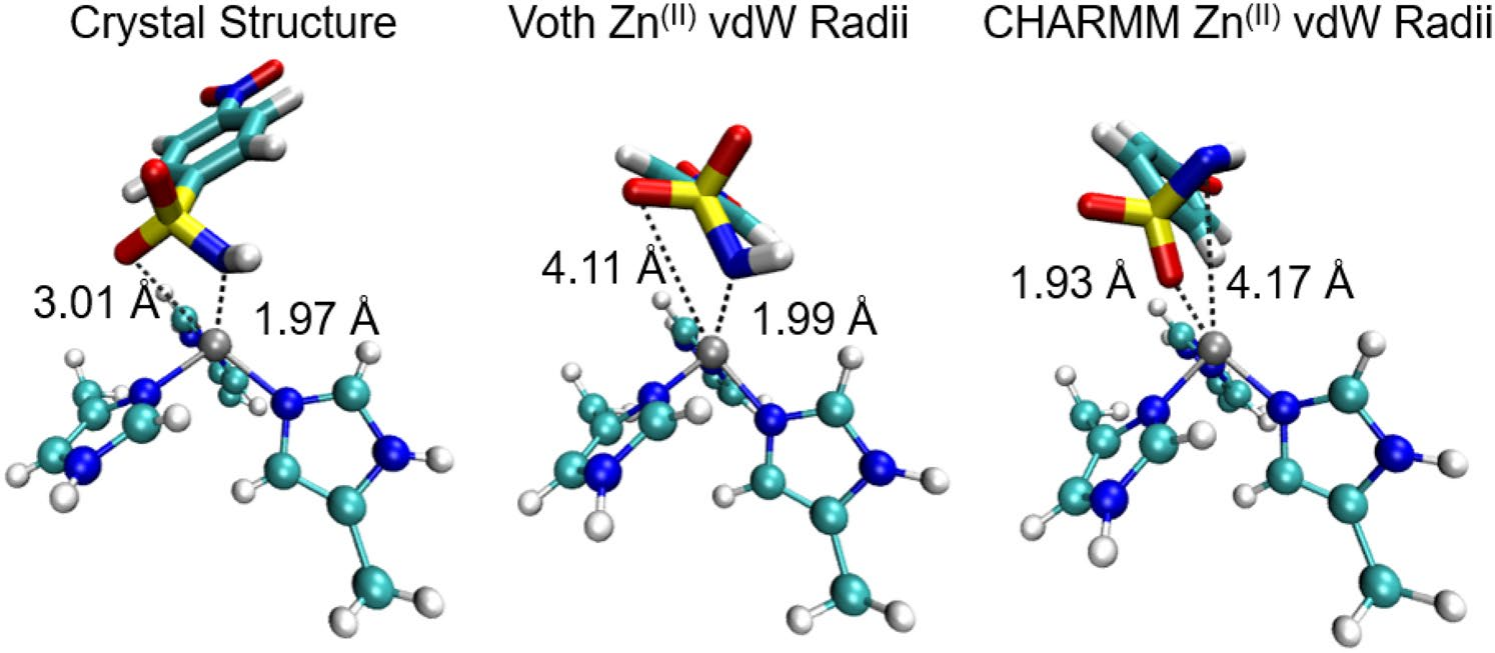
Representative binding pose of Ligand **4** using different Lennard–Jones parameters. Crystal structure (PDB ID 6RH4, left), Snapshots from a simulation of the solvated protein–ligand system with the zinc(II) vdW radii optimised by Lu and Voth (centre), and snapshot from a simulation of the solvated protein–ligand system with the CHARMM zinc(II) vdW radii (right). Only ligand and Zn(His)_3_ binding site are depicted. Average interatomic distances over a 4 ns production run are shown

**Fig. 6:**
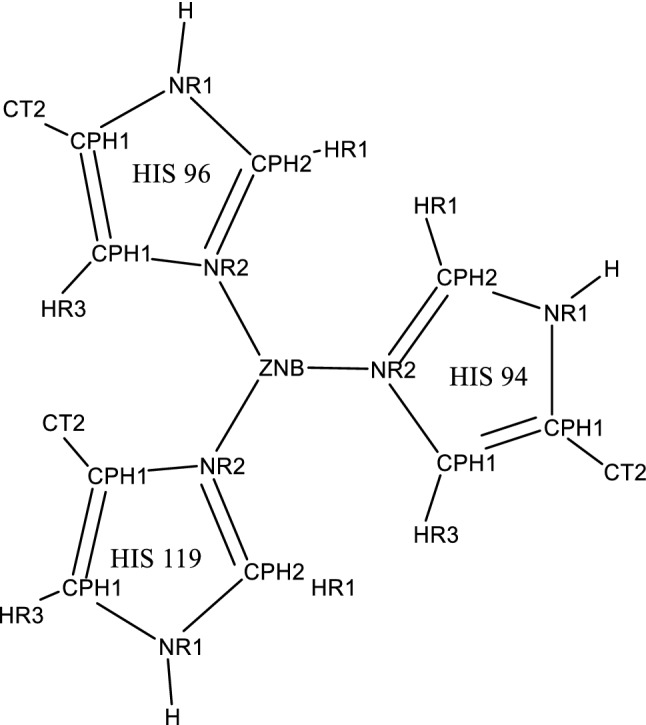
Atom typing for Zn(II) and coordinating histidine residues

Lin and Wang systematically derived bonded parameters for zinc containing systems in the AMBER force field [40]. Similarly, Lu and Voth also developed bonded parameters defining the Zn(His)_3_ complex for carbonic anhydrase [43]. These bonded parameters were used in a partially bonded model in work within the Meuwly group [65]. In this approach, bonds are defined linking each of the three histidine nitrogen atoms to the zinc atom, whereas the bond between the zinc and sulfonamide nitrogen is retained using collective variables and there was a moderately strong correlation between their MM/GBSA and experimental binding energies [21]. Inspired by these efforts, a similar partially bonded model was applied here. As the choice of zinc Lennard–Jones parameters can affect the coordination in the active site [48], two different vdW parameters were tested: the default CHARMM Zn^2+^ Lennard-Jones parameters, and one published Lu and Voth. In the latter, the ε_min_ (well depth at the minimum) is smaller than the parameter in the CHARMM forcefield, and so the attractive force due to the interaction is smaller. The use of the CHARMM Lennard–Jones parameters led to ligands adopting an incorrect coordination geometry, where the sulfonamide nitrogen is displaced by a sulfonamide oxygen in the coordination shell, with the trajectory-averaged distance between the zinc and sulfonamide nitrogen of 4.17 Å, which is 2.2 Å greater than found in the crystal structure. (Fig. 5). The use of the Zn(II) Lennard–Jones parameters optimised by Lu and Voth prevents this change in binding geometry and hence was used for all subsequent simulations [43]. It is clear that regardless of the choice of zinc vdW parameter, the ligand loses its bidentate binding mode, and instead retains a monodentate interaction with the zinc. The trajectory-averaged zinc–sulfonamide oxygen distance is 1.1 Å larger than the crystal structure for simulations with the Voth Zn (II) parameter. The zinc–histidine bond distance was restrained to a distance of 2.0 Å to better reproduce crystal structure geometries. A complete list of parameters can be found in Table 1 and Fig. 6.

**Table 1:** Bonded and non-bonded parameters used to maintain the zinc tetrahedral geometry in conjunction with the CHARMM force field. Adapted from the work of Lu and Voth [43], and the Meuwly group [65]. Atom typing shown in Fig. 6

Bonded parameter	Equilibrium value	Force constant	
Bonds	Equilibrium distance/Å	*k*/kcal mol^−1^ Å^−1^	
ZNB – NR2	2.0	40.0	
Angles	Equilibrium angle/°	*k*/kcal mol^−1^ rad^−2^	
NR2 – ZNB – NR2	109.5	23.0	
CPH2 – NR2 – ZNB	126.0	20.0	
Dihedrals	Equilibrium angle/°	Multiplicity	*k*/kcal mol^−1^
NR1 CPH2 NR2 ZNB	180.0	2	5.0
CPH1 CPH1 NR2 ZNB	180.0	2	5.0
HR1 CPH2 NR2 ZNB	180.0	2	5.0
HR3 CPH1 NR2 ZNB	180.0	2	5.0
Improper dihedrals	Equilibrium angle/°	Multiplicity	*k*/kcal mol^−1^ rad^−2^
ZNB NR2 NR2 NR2	0.0	0	25.0
Nonbonded parameters	Charge/au	ε	*R*_min_
ZNB^a^	1.0	0.014	1.0
Collective variables	Equilibrium angle/°	*k*/kcal mol^−1^ (units)^−2^	
N_S_ Zn N_HIS_	120	0.05	
S_S_ N_S_ Zn N_HIS_	Crystal structure (PDB ID 4YX4)	0.00111	

^a^CHARMM Zn FF parameter: charge =  + 2.0, ε = 0.25, *R*_min_ = 1.09

**Fig. 7:**
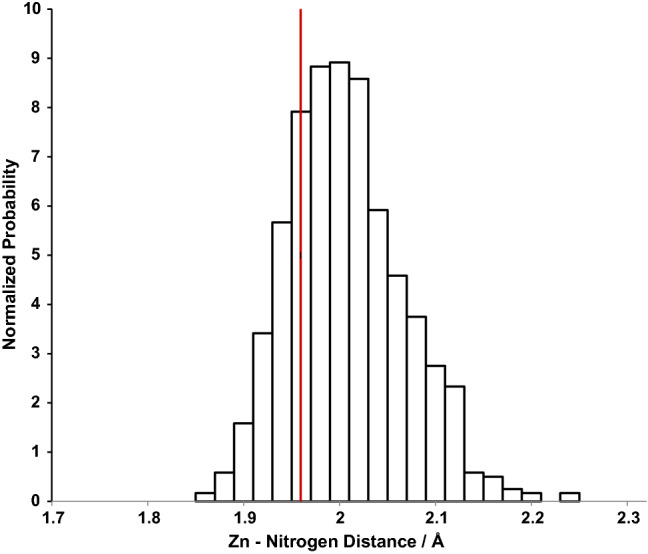
Normalized histogram of the distance between the zinc and sulfonamide nitrogen over an 8 ns trajectory for Ligand **4**. Red line represents the crystal structure distance of 1.97 Å (PDB ID 6RH4)

The distribution of Zn-N(sulfonamide) distance along a MD trajectory for CAII and ligand **4** is plotted in Fig. 7 which coincides reasonably well with the crystal structure distance.

### Effect of QM ligand charges on MM/GBSA binding energies

**Fig. 8:**
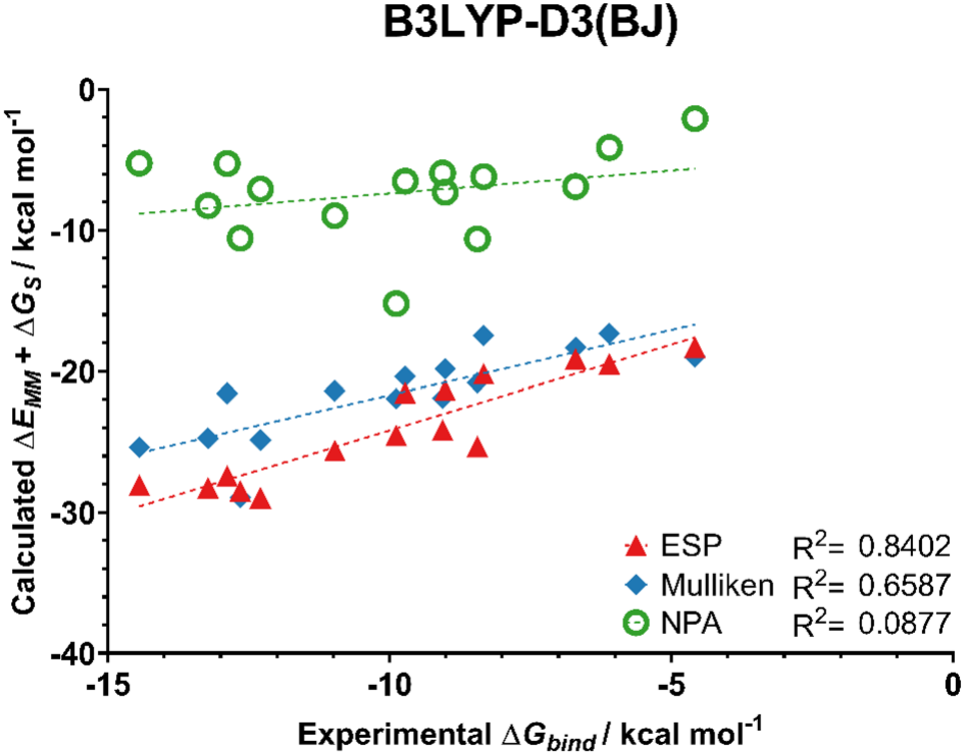
Predicted binding affinities of ligands **1**–**15** from MM/GBSA simulations with ESP, Mulliken, and NPA charges from a B3LYP-D3(BJ)/6-31G(d,p) calculation on the ligands. MM/GBSA energies averaged from 5 independent 4 ns production runs

**Fig. 9:**
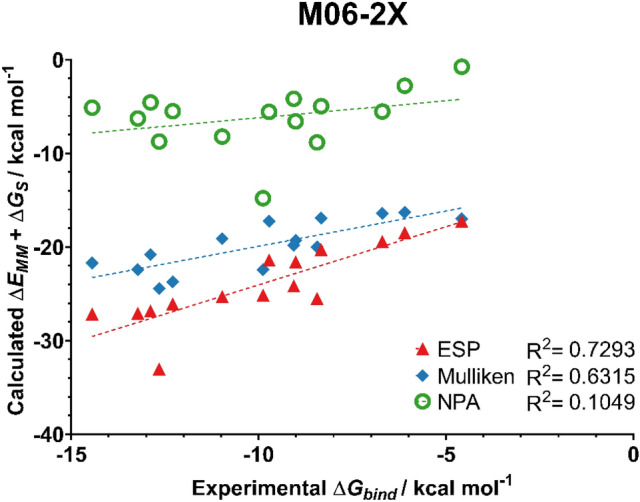
Predicted binding affinities of ligands **1**–**15** from MM/GBSA simulations with ESP, Mulliken, and NPA charges from an M06-2X/6-31G(d,p) calculation on the ligands. MM/GBSA energies averaged from 5 independent 4 ns production runs

**Fig. 10:**
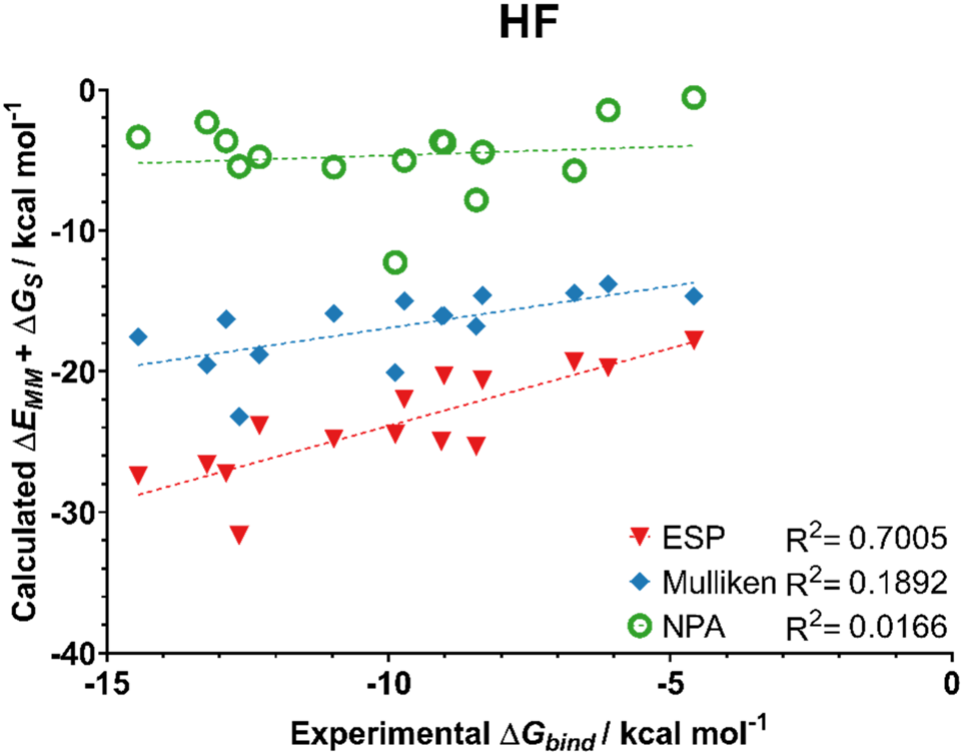
Predicted binding affinities of ligands **1**–**15** from MM/GBSA simulations with ESP, Mulliken, and NPA charges from a HF/6-31G(d,p) calculation on the ligands. MM/GBSA energies averaged from 5 independent 4 ns production runs

Having established a suitable set of force field parameters, this section focuses on conducting MD simulations using different atomic charges to represent the ligand and the effect this has on the resulting MM/GBSA binding energies. Atomic charges were calculated using the Mulliken, ESP, and NPA schemes at the HF/6-31G(d,p), B3LYP-D3(BJ)/6-31G(d,p), and M06-2X/6-31G(d,p) levels of theory. These charges were compared for their ability to predict trends in binding affinities for a set of 15 ligands previously studied in the Meuwly group (ligands **1**–**15** from Fig. 2) [65]. In that work, a correlation coefficient of 0.55 was achieved for these 15 ligands which is somewhat lower compared to the values obtained in this work (vide infra). The effect of atomic charges on the binding affinity is compared in Figs. 8, 9 and 10.

For this set of ligands, the use of ESP atomic charges consistently gave the best correlation between the experimental and MM/GBSA binding affinities. Notably, ESP charges calculated at the B3LYP-D3(BJ)/6-31G(d,p) level of theory yielded very good correlation with the experimentally derived binding energies, with an R^2^ value of 0.84.

Mulliken charges also yielded good correlation with experimental data when calculated with the two DFT methods. In particular, the protocol used here in conjunction with charges obtained at the B3LYP-D3(BJ) level of theory showed an improved performance compared to that of Meuwly and co-workers (R^2^ = 0.55 c.f. 0.66 in this work). Compared to ESP charges, Mulliken charges are not suitable for the prediction of trends in binding affinities when computed at the HF level of theory. This is unsurprising, as Mulliken charges are known to be sensitive to the level of theory at which they are obtained [39].

**Scheme 1:**
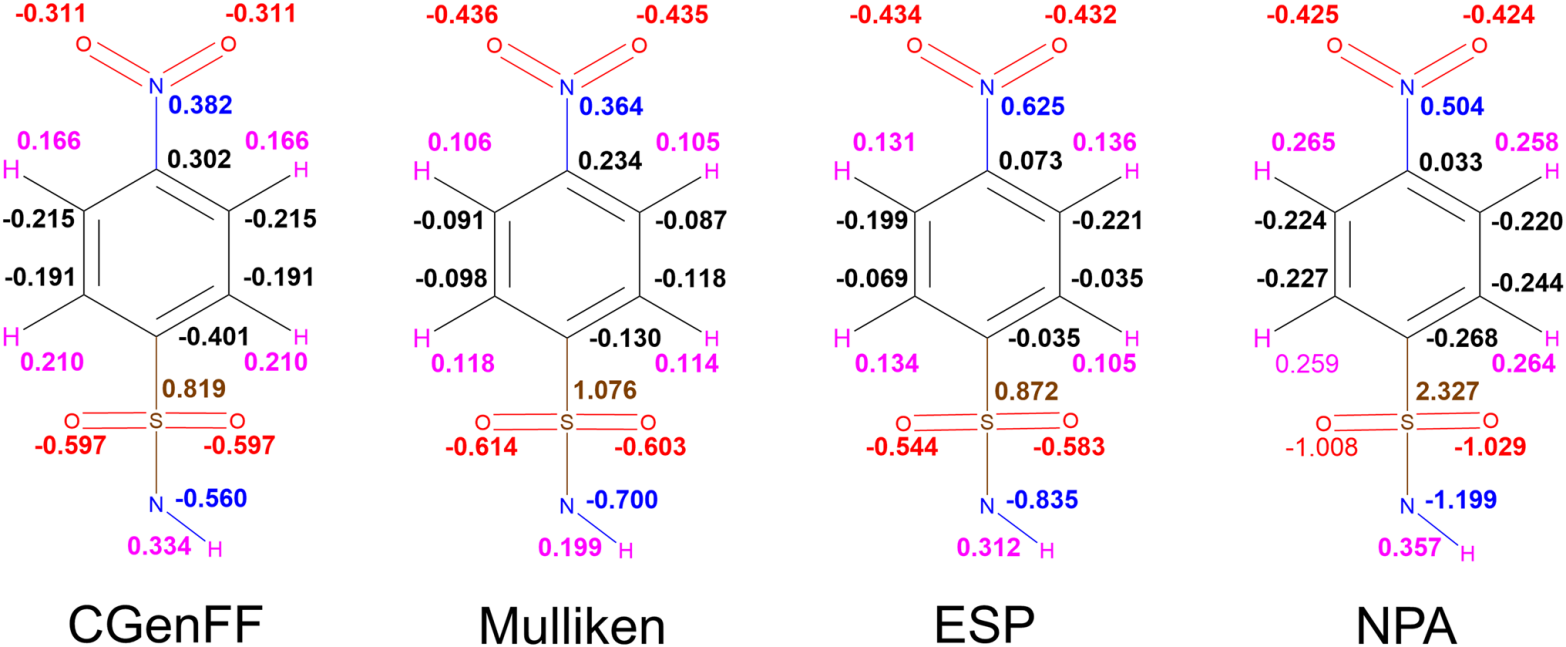
Distribution of partial charges of ligand **4** derived from CGENFF and B3LYP-D3(BJ)/6-31G(d,p) geometry optimisations and calculations. Partial charges obtained from Mulliken, ESP or NPA fittings. Partial charges rounded to three decimal places

**Fig. 11:**
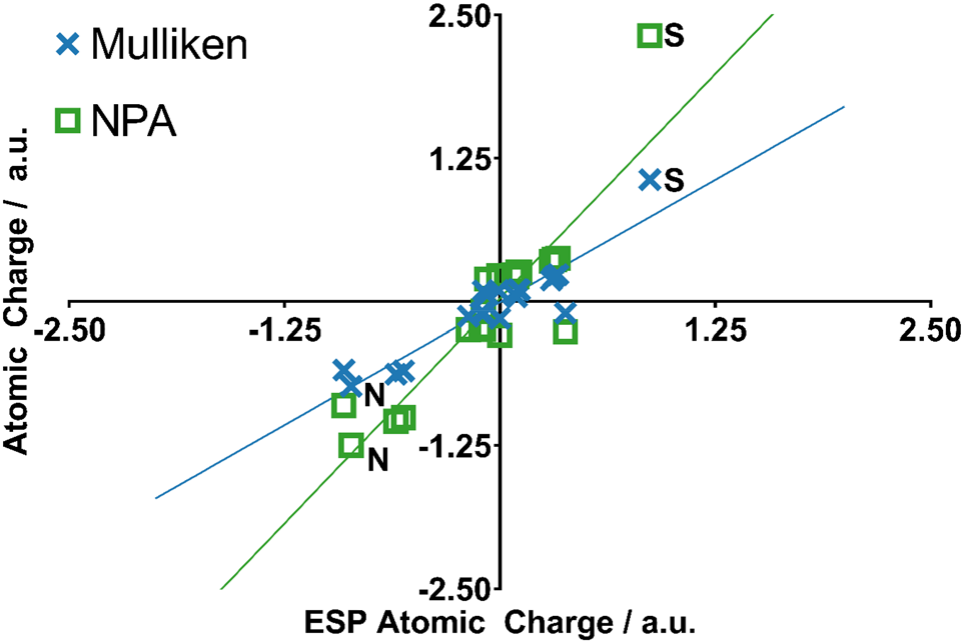
Mulliken and NPA charges plotted against ESP charges for all atoms in all ligands. NPA charges are significantly more polarised than Mulliken charges

Despite previous work demonstrating NPA charges can accurately model solute–solvent interactions [14], here the use of NPA charges gave no correlation with the experimental data irrespective of the choice of QM method. Scheme 1 compares the atomic charges obtained using different schemes for a representative ligand **4**. The automatically assigned CGENFF atomic charges are also shown for comparison but were not considered in the MD simulations due to the high penalty scores associated with these assigned charges. As shown, the use of NPA charges significantly polarises the charges around the sulfonamide head. In particular, the charge on the sulfur atom is around 2.5 times greater when assigned using NPA charges compared to ESP charges. Figure 11 shows the correlation between Mulliken and ESP compared to the correlation between Mulliken and NPA charges. It is clear that the NPA charge scheme yields charges that are disproportionately larger in magnitude compared to Mulliken charges, most notably for the sulfonamide S atom, which sit in the top right of the graph, and the sulfonamide nitrogen at the bottom left.

**Fig. 12:**
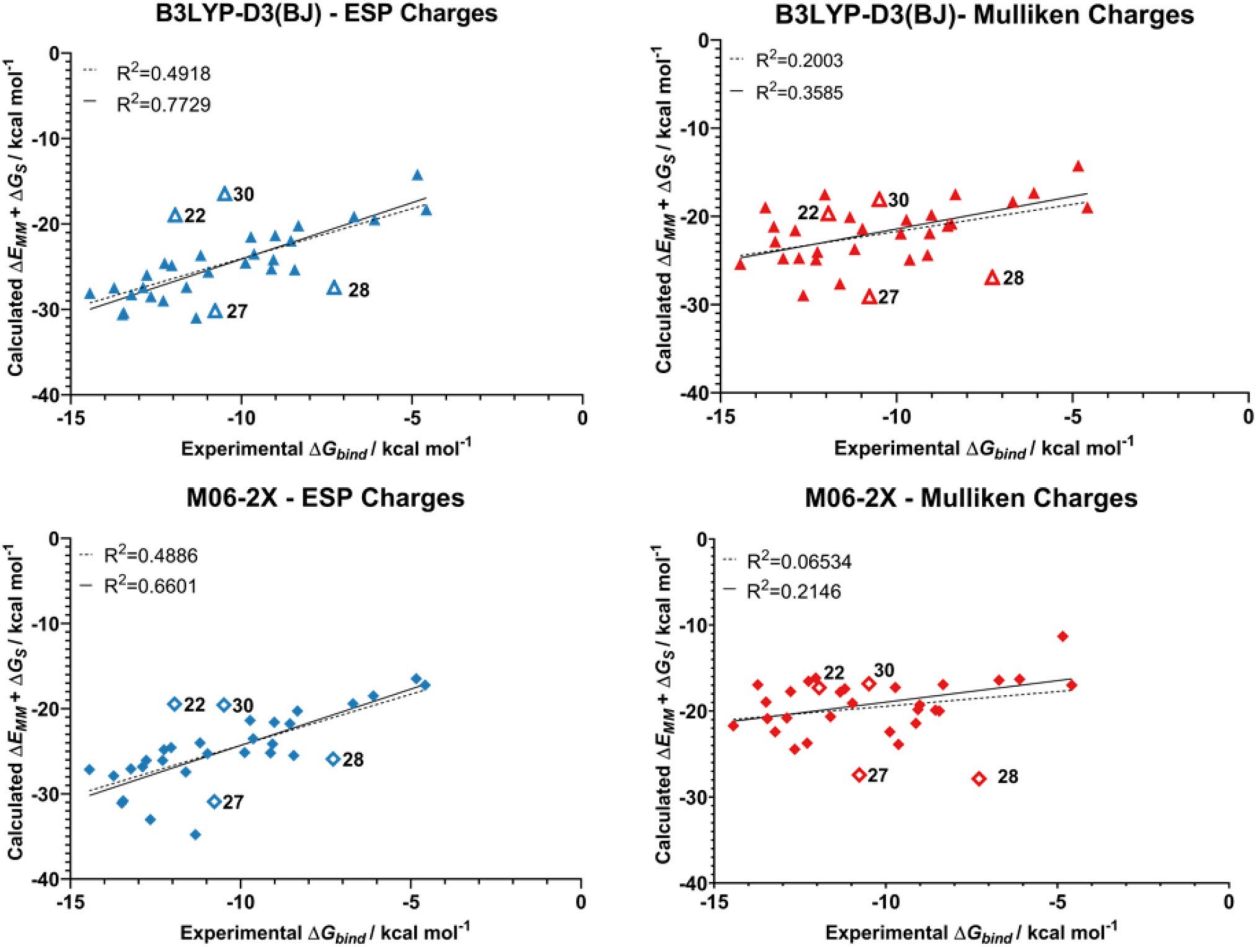
MM/GBSA binding affinities of ligands **1**–**32** calculated with ESP or Mulliken atomic charges from DFT methods. Hollow symbols denote outliers, and dashed lines the correlation including the outliers. MM/GBSA energies averaged from 5 independent 4 ns production runs

To test the broader performance of the best performing charge schemes, we expanded on the test set to include a total of 32 chemically diverse ligands (Fig. 2). Binding affinities were predicted from simulations using Mulliken or ESP charges from either B3LYP-D3(BJ) or M06-2X, and compared to experimental data. The binding affinities and uncertainty are presented in Table 2, and plotted in Fig. 12. The RMSD in predicted absolute binding energies and pairwise RMSD in relative binding energies are also presented in Table 2, where the latter is about 2–3 times smaller presumably due to systematic cancellation of errors.

**Table 2:** Predicted MM/GBSA Binding Affinities of ligands **1** to **32** to hCA II with different charge schemes. Binding data is the average of 5 independent 4 ns trajectories. Uncertainty presented as 2 times the standard error in the mean

Ligand	Experimental Δ*G*_*bind*_/kcal mol^−1^	B3LYP−D3(BJ)		M06−2X	
		ESP Δ*E*_*MM*_ + Δ*G*_*s*_/kcal mol^−1^	Mulliken Δ*E*_*MM*_ + Δ*G*_*s*_/kcal mol^−1^	ESP Δ*E*_*MM*_ + Δ*G*_*s*_/kcal mol^−1^	Mulliken Δ*E*_*MM*_ + Δ*G*_*s*_/kcal mol^−1^
1	− 8.33	− 20.19 ± 0.48	− 17.48 ± 0.66	− 20.28 ± 0.33	− 16.92 ± 0.47
2	− 9.72	− 21.51 ± 0.27	− 20.38 ± 1.03	− 21.38 ± 0.22	− 17.25 ± 1.30
3	− 6.7	− 19.13 ± 0.44	− 18.32 ± 1.52	− 19.41 ± 0.82	− 16.42 ± 1.43
4	− 9.88	− 24.54 ± 0.50	− 21.94 ± 1.10	− 25.15 ± 0.42	− 22.41 ± 0.40
5	− 6.1	− 19.48 ± 0.74	− 17.33 ± 1.12	− 18.50 ± 1.04	− 16.30 ± 0.37
6	− 9.01	− 21.34 ± 0.48	− 19.82 ± 0.36	− 21.60 ± 0.35	− 19.29 ± 0.53
7	− 10.97	− 25.61 ± 0.43	− 21.39 ± 1.16	− 25.28 ± 0.78	− 19.09 ± 0.82
8	− 12.29	− 28.97 ± 2.15	− 24.87 ± 2.39	− 26.08 ± 3.91	− 23.73 ± 2.03
9	− 12.65	− 28.49 ± 2.74	− 28.94 ± 2.18	− 33.02 ± 2.94	− 24.44 ± 2.45
10	− 12.88	− 27.42 ± 0.29	− 21.57 ± 1.23	− 26.83 ± 0.48	− 20.80 ± 0.55
11	− 13.22	− 28.26 ± 1.67	− 24.75 ± 1.35	− 27.07 ± 1.16	− 22.41 ± 2.25
12	− 14.44	− 28.08 ± 1.37	− 25.38 ± 0.62	− 27.15 ± 1.15	− 21.71 ± 1.13
13	− 8.44	− 25.34 ± 0.59	− 20.81 ± 1.37	− 25.49 ± 0.79	− 19.99 ± 0.60
14	− 4.58	− 18.31 ± 0.37	− 18.98 ± 0.83	− 17.23 ± 0.89	− 16.99 ± 0.54
15	− 9.06	− 24.16 ± 0.43	− 21.90 ± 0.61	− 24.14 ± 0.47	− 19.82 ± 0.81
16	− 11.61	− 27.38 ± 0.85	− 27.63 ± 0.47	− 27.43 ± 0.91	− 20.65 ± 1.39
17	− 12.25	− 24.58 ± 1.07	− 24.03 ± 1.32	− 24.82 ± 1.40	− 16.53 ± 1.58
18	− 4.84	− 14.20 ± 1.60	− 14.24 ± 1.24	− 16.48 ± 0.65	− 11.31 ± 0.46
19	− 11.19	− 23.67 ± 1.01	− 23.71 ± 0.50	− 24.02 ± 1.08	− 17.41 ± 0.75
20	− 12.77	− 25.96 ± 0.89	− 24.70 ± 0.69	− 26.07 ± 1.03	− 17.73 ± 1.73
21	− 8.55	− 21.96 ± 0.28	− 21.07 ± 0.30	− 21.77 ± 0.43	− 19.89 ± 0.37
22	− 11.94	− 18.96 ± 0.52	− 19.68 ± 0.87	− 19.46 ± 0.23	− 17.29 ± 0.69
23	− 13.45	− 30.37 ± 2.09	− 22.86 ± 1.63	− 30.82 ± 0.60	− 20.88 ± 1.36
24	− 13.73	− 27.46 ± 0.48	− 18.97 ± 1.11	− 27.89 ± 0.93	− 16.94 ± 0.74
25	− 11.39	− 36.19 ± 1.37	− 24.01 ± 2.02	− 38.18 ± 1.16	− 23.06 ± 0.81
26	− 9.63	− 23.49 ± 0.36	− 24.90 ± 0.76	− 23.53 ± 1.51	− 23.88 ± 1.50
27	− 10.77	− 30.16 ± 0.43	− 29.10 ± 0.75	− 30.91 ± 0.86	− 27.42 ± 0.46
28	− 7.28	− 27.43 ± 3.09	− 26.93 ± 2.05	− 25.91 ± 2.55	− 27.87 ± 2.98
29	− 9.12	− 25.25 ± 0.36	− 24.37 ± 0.67	− 25.20 ± 1.00	− 21.43 ± 0.89
30	− 10.49	− 16.46 ± 3.33	− 18.09 ± 2.35	− 19.56 ± 0.61	− 16.82 ± 2.88
31	− 13.49	− 30.59 ± 1.49	− 21.15 ± 1.66	− 31.08 ± 1.51	− 18.95 ± 1.81
32	− 12.04	− 24.84 ± 0.94	− 17.50 ± 0.46	− 24.56 ± 0.87	− 16.16 ± 0.62
Pairwise RMSD^*a*^		4.43	4.76	4.59	5.36
RMSD^*b*^		14.45	14.73	12.03	9.91

^a^Pairwise RMSD refers to the RMSD in all pairwise relative binding energies (there are 496 or ^32^C_2_ of these)

^b^RMSD refers to the RMSD in the absolute binding energies (32 data points)

Notably, for this larger and more diverse dataset the correlation between experimental and predicted binding affinities reduced significantly compared to the original set containing ligands **1**–**15**. This effect was independent of the charge scheme used. ESP charges showed better correlation with the experimental data than Mulliken charges regardless of the QM level of theory. Both the B3LYP-D3(BJ) and M06-2X charges gave very similar correlation coefficients (R^2^ = 0.49 and 0.48 respectively). Nevertheless, when four outliers (**22**, **27**, **28**, and **30**) were removed, this restored the good correlation with experimental binding affinities where the R^2^ increased to 0.77 in the best case for B3LYP ESP charges.

Further inspection of the four outliers reveals several interesting observations. As with the docking protocol, **22** (trifluomethylsulfonamide) has a significantly lower pK_*a*_ (6.3) than the other ligands in the dataset, and hence the energetic cost of deprotonation is lower [37]. The pK_*a*_ values of the other ligands range from 7.4 to 10.8, with the majority above 8.5. At a pH of 7, the difference in deprotonation energy between a ligand with a pK_*a*_ of 6.3 and 8.3 translates to an approximately 2 kcal mol^−1^ change in binding free energy. Consequently, whilst this effect likely cancels for the other ligands with similar pK_*a*_ values, the lower energetic requirement for deprotonation resulted in an underestimation of the binding affinity of **22** relative to other ligands and should bring it closer to the trend lines when the effect is included (Fig. 12).

Ligand **28**, which contains a carbohydrate group has consistently underestimated binding affinities. This appears to be consistent with literature where the CHARMM carbohydrate forcefield is known to significantly and consistently underestimate protein—carbohydrate binding affinities [59].

**Fig. 13:**
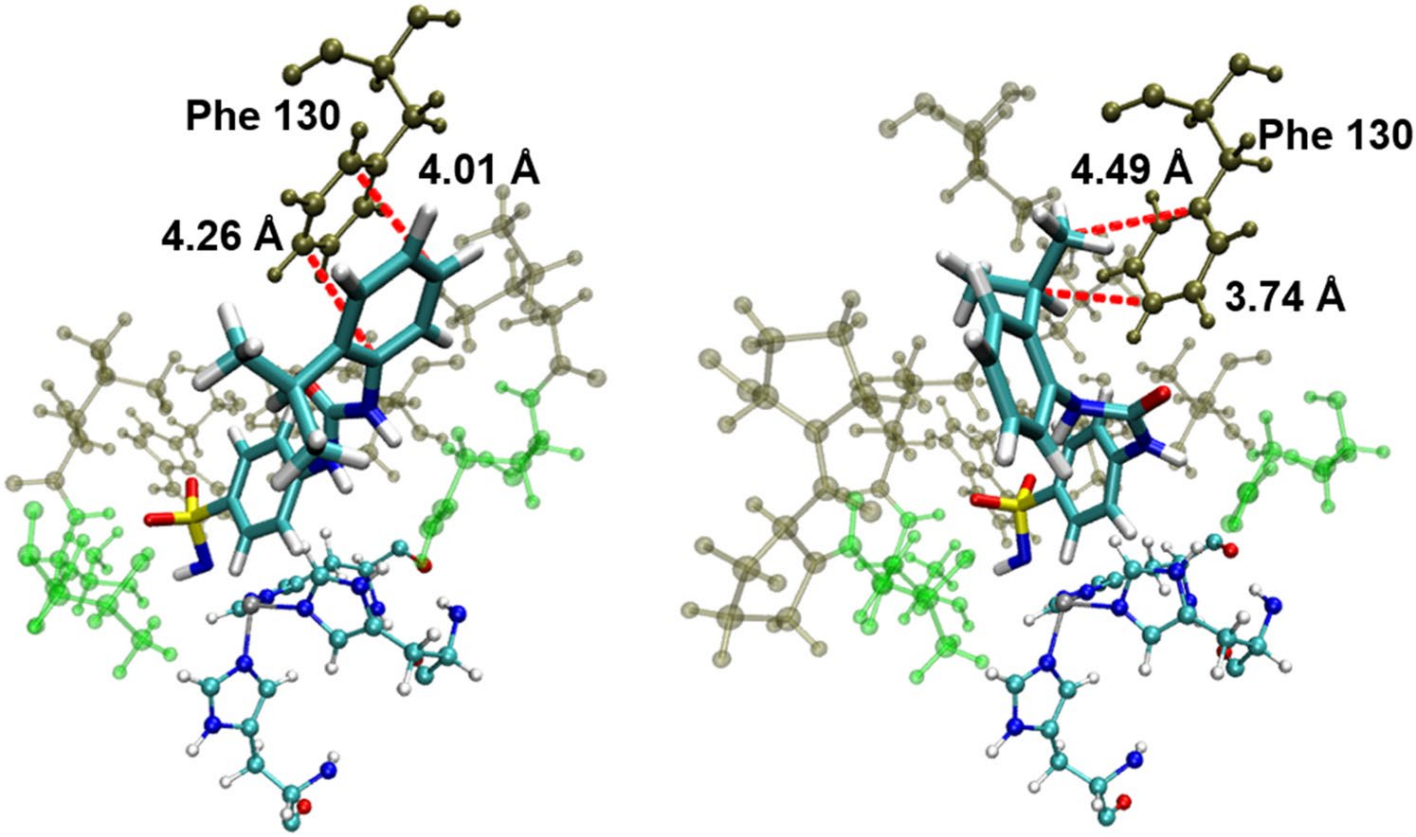
Representative binding modes of **28** in two different simulations. The ureido group can adopt different conformations, which affects the position of the isopropyl group. The *anti-anti* (left) and *syn-anti* (right) conformations are observed. Protein residues shown coloured by their residue type. Non-polar residues shown in brown, polar residues shown in green. Hydrophobic carbon–carbon interaction distances indicated by red dashed lines

Whilst ligands **26**–**29** all had overestimated binding affinities, ureidobenzenesulfonamides **27** and **28** consistently showed the largest deviation from the trendline. These four ligands include SLC-0111 (**26**) and its derivatives which are more conformationally flexible than the majority of the ligands in the test set. Specifically, these compounds can adopt 3 distinct conformations, namely *syn-syn*, *syn-anti*, and *anti-anti*, corresponding to different orientations of the -NH groups pointing either towards (syn) or away from (anti) the chalcogen. For the ureidobenzensulfonamides **26**, **28** and **29**, different binding poses are observed in independent trajectories, corresponding to the *anti-anti* and *syn-anti* conformers. Whilst **26** and **29** have similar binding affinities for each pose, the *ortho*-substituted isopropyl phenyl group in **28** is significantly affected by the orientation of the urea as shown in Fig. 13. Binding in the *anti-anti* conformer results in a 7 kcal mol^−1^ increase in binding affinity relative to the *syn-anti*. In the *syn-anti* pose the isopropyl group points towards the hydrophobic pocket, whereas in the *anti-anti* conformer the phenyl ring is more easily able to form a strong hydrophobic interaction with a phenylalanine residue. This large variance results in the large uncertainties as shown in Table 2, with an uncertainty of 3.06 kcal mol^−1^ in MM/GBSA binding energies obtained from B3LYP-D3(BJ) ESP charges. To examine if this was due to insufficient sampling, 100 simulations were run with B3LYP-D3(BJ) ESP charges (50 with an *anti-anti* starting pose and 50 with a *syn-syn* starting pose). The binding affinity was predicted to be -25.6 kcal mol^−1^ (σ = 2.44) compared with -27.43 kcal mol^−1^ (σ = 3.46) when only five trajectories were run. This 2 kcal mol^−1^ shift brings the data point closer to the trend line indicating that insufficient sampling is likely an issue here. Notably, regardless of starting pose, trajectories containing both the *syn-syn* and *syn-anti* conformers were observed. The crystal structure shows that ligand **28** binds in the *anti-anti* conformation [53]. For the thiourea **27**, the ligand adopts the *syn-syn* conformer in all trajectories, whilst the crystal structure shows the ligand binds in a *syn-anti* conformer [2]. However, fixing the ligand in the crystal structure conformer may not necessarily improve the correlation with experiment, as evidenced by the crystal structure *anti*-*anti* conformer of ligand **28** having a greater divergence from the predicted trendline of binding affinities.

When these ligands are excluded, the use of B3LYP-D3(BJ) optimised ESP charges has a strong correlation with the experimental binding affinities (R^2^ = 0.77), which only slightly lower than the correlation observed with only the original 15 ligands. Similarly, ESP charges obtained with M06-2X also show reasonable correlation, with an R^2^ of 0.66. Mulliken charges show limited correlation between the predicted and experimental binding data even with these difficult to model inhibitors excluded.

## Conclusions

This work evaluates methods for predicting relative binding affinities for a set of structurally diverse ligands. The AutoDock4_Zn_ docking program showed a moderately strong ability to rank ligands (R^2^ = 0.64). However, this method was unreliable in reproducing the crystal structure binding pose of a set of 24 ligands, with a mean RMSD of 6.05 Å. The more theoretically robust MM/GBSA method was shown to improve the correlation between predicted and experimental binding affinities when ESP ligand charges were used. Simulations with charges obtained from DFT optimisations at the B3LYP-D3(BJ)/6-31G(d,p) level of theory showed the strongest correlation with the experimental values (R^2^ = 0.77). When applied to a subset of arylsulfonamides used in a previous study [65], this approach shows an excellent correlation of R^2^ = 0.84. Similarly, M06-2X/6-31G(d,p) ESP charges had a moderately strong correlation (R^2^ = 0.66) over the whole dataset. However, the use of other charge schemes showed limited to no correlation. Mulliken charges showed poorer agreement with the experimental binding data regardless of level of theory, with correlations of R^2^ = 0.36 and R^2^ = 0.21 for B3LYP-D3(BJ)/6-31G(d,p) and M06-2X/6-31G(d,p) charges respectively. Finally, NPA charges showed no ability to rank ligands by binding affinity. Overall, the results presented here demonstrate that MM/GBSA can be used to evaluate ligand binding affinities provided that a validated set of parameters is used.

## Supplementary Information

Below is the link to the electronic supplementary material.

Supplementary file1 (ZIP 63 kb)

Supplementary file2 (XLSX 141 kb)

Supplementary file3 (PDF 1755 kb)

## Data Availability

All data generated and/or analysed during this study are included in this published article and its supplementary information files.
