# Supplementary material for: MM/GBSA prediction of relative binding affinities of carbonic anhydrase inhibitors: effect of atomic charges and comparison with Autodock4Zn
Source: J Comput Aided Mol Des. 2023 Mar 17;37(4):167–82. doi: 10.1007/s10822-023-00499-0 (PMC10050039; doi:10.1007/s10822-023-00499-0)
Supplement: Supplementary file 3 — Supplementary file3 (PDF 1755 kb) [file 10822_2023_499_MOESM3_ESM.pdf]

MM/GBSA Prediction of Relative Binding Affinity of Carbonic Anhydrase Inhibitors – Effect of Atomic Charges and Comparison with Autodock4$_{Zn}$

Mackenzie Taylor and Junming Ho*

School of Chemistry, University of New South Wales, NSW 2052, Australia

Supporting Information

Table of Contents

Figure S1. Effect of pKa corrections on docked binding affinities	p. S2
Figure S2. Effect of pKa corrections on MM-GBSA binding affinites	p. S3

* Correspondening author email: junming.ho@unsw.edu.au

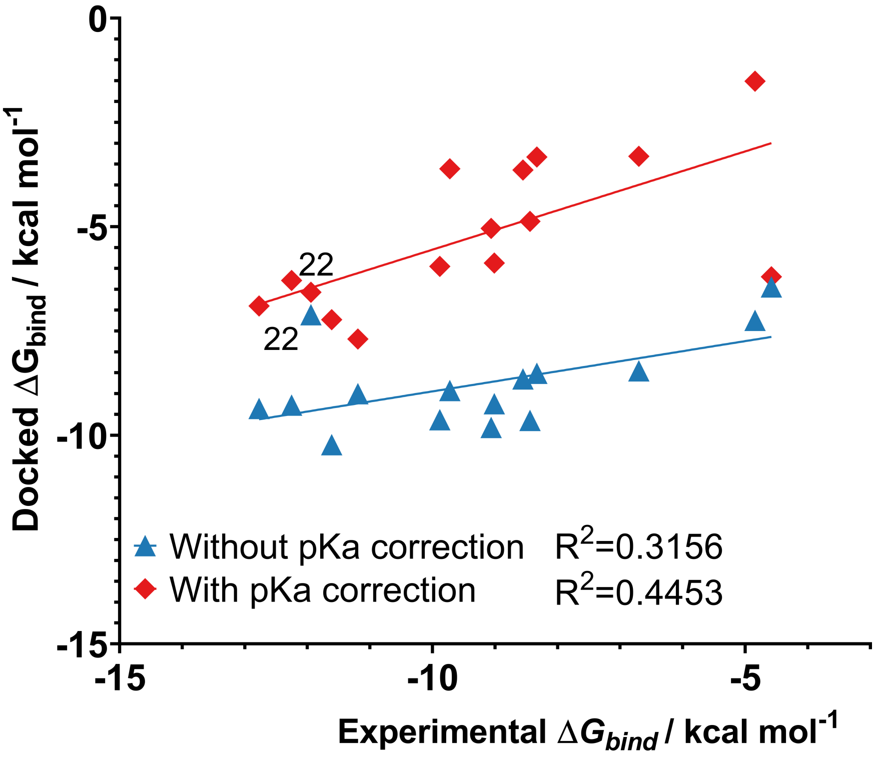

Figure S1 – Predicted binding affinity of the full dataset of ligands by docking with the AutoDock4$_{Zn}$ force field in AutoDock Vina both with (red) and without (blue) a correction for the ligand deprotonation energy.

The pKa correction is evaluated through the following equations (Chem. Rev. 2008, 108, 946).

$$\Delta G = -RT\ln(1/K_d) = RT\ln K_d$$

$$K_d^{corrected} = \frac{K_d^{calc}}{\theta_{NH^-}\theta_{CA}}$$

$$\theta_{NH^-} = [1 + 10^{pK_a(Sulfonamide-NH_2)-pH}]^{-1}$$

$$\theta_{CA} = [1 + 10^{pH - pK_a(CA-Zn^{II}-OH_2^+)}]^{-1}$$

Where ΔG is the Gibbs free energy of binding, $K_d^{corrected}$ and K_d^{Calc} are the p*K*a corrected and predicted dissociation constants respectively, pKa(sulfonamide-NH_2) is the pKa of the ligand, the pH is the pH of the protein environment, here taken as 6.5. The pKa(CA-Zn^{II}-OH_2^+) is the pKa of the water coordinated zinc binding site, taken as 6.8 (Chem. Rev. 2008, 108, 946).

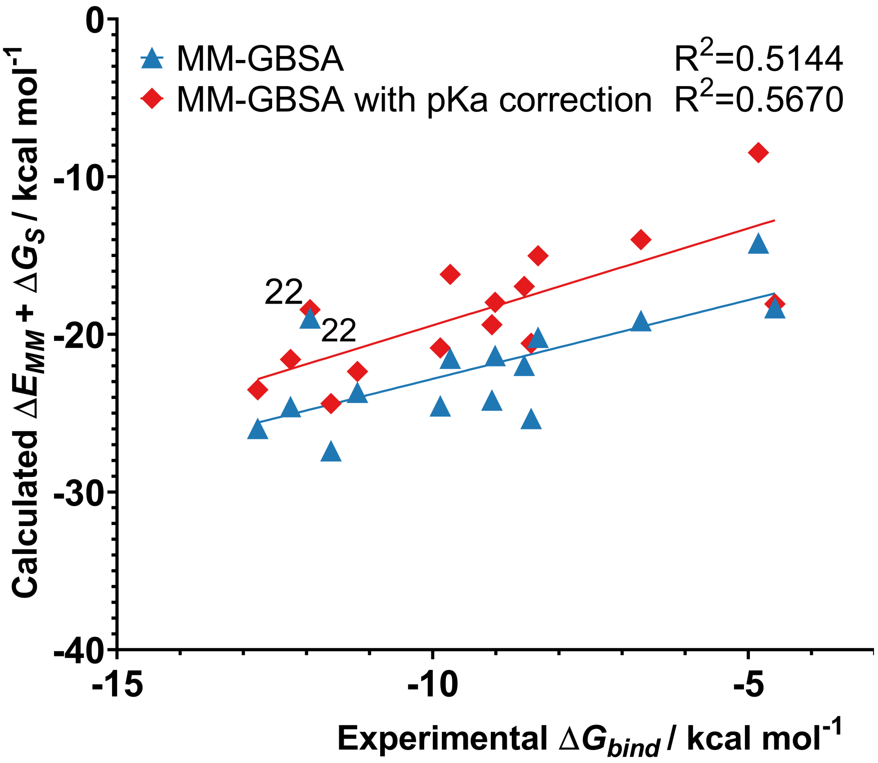

Figure 2 - Predicted binding affinity of the full dataset of ligands by MM/GBSA simulations with B3LYP-D3(BJ) ESP charges. Binding affinity calculated both with (red) and without (blue) a correction for the ligand deprotonation energy. MM/GBSA energies averaged from 5 independent 4 ns production runs.
